# Effectiveness of Moxibustion Combined with Chinese Medicine in the Treatment of Spleen and Stomach Deficiency Cold-Type Gastroparesis: A Meta-Analysis of Randomized Controlled Trials

**DOI:** 10.1155/2022/6552819

**Published:** 2022-11-10

**Authors:** Qingwen Gan, Zerong Lian, Lilan Zheng, Qifan Feng, Lihua Wei, Ying Wang

**Affiliations:** ^1^School of Nursing, Nanchang University, Nanchang 330006, China; ^2^Heping Hospital Affiliated to Changzhi Medical College Hospital Affiliated to Changzhi Medical College, Changzhi 046000, China; ^3^The First Affiliated Hospital of Nanchang University, Nanchang 330000, China

## Abstract

**Objective:**

To investigate the effectiveness of moxibustion combined with Chinese medicine in the treatment of spleen and stomach deficiency cold-type gastroparesis by meta-analysis and to provide the clinical basis for its treatment.

**Methods:**

A computer search of eight databases was performed for published and unpublished randomized controlled trials on moxibustion for spleen and stomach deficiency cold-type stomach pain from domestic and international clinical trial centers. The study was divided into a combination of a moxibustion and Chinese medicine group and a regular Western medicine group, and the outcome indicators were “effective” and “ineffective.” The relative risk (RR) was used as the effect indicator for the dichotomous data, and the meta-analysis was performed using Reviewer Manager 5.4 and Stata17.0 software.

**Results:**

A total of 8 randomized controlled studies with 729 patients were included, and all studies were analyzed for comparability of patients' baseline information, with no statistically significant differences found (*P* > 0.05). The meta-analysis results showed that the pooled effect size RR for the eight studies was 1.24 (95% confidence interval 1.16–1.32), and the statistical significance test was *Z* = 6.69 (*P* < 0.05), indicating that the difference was statistically significant.

**Conclusion:**

The meta-analysis concluded that the efficacy of moxibustion combined with Chinese medicine for the treatment of spleen and stomach deficiency cold-type gastroparesis was superior to that of regular Western medicine, but more high-quality studies are needed to confirm this finding.

## 1. Introduction

Currently, the combination of various factors, such as the fast pace of modern life, irregular diet, overeating, staying up late, anxiety, and stress, has led to an increased incidence of spleen and stomach disorders [1]. Globally, more than half of the population currently suffers from spleen and stomach disorders. According to the World Health Organization, the prevalence of gastrointestinal diseases in the population is as high as 80% and is increasing at an annual rate of 17.43%, with 120 million patients suffering from spleen and stomach diseases in China. The incidence of stomach diseases is gradually increasing in younger people [2], which seriously affects their quality of life. Among the spleen and stomach diseases, stomach pain with spleen and stomach deficiency cold-type is one of the most common clinical diseases [3]. Chinese medicine theory considers that [4] the spleen and the stomach are in the same place as each other and share the function of transporting nutrients in food. The spleen transports nutrients upward to the heart, lungs, head, and eyes to produce qi and blood to nourish the whole body through the action of the heart and lungs, while the stomach digests food and transports it down to the intestines. The stomach is dependent on the spleen's transport ability, so stomach diseases often involve the spleen, and spleen diseases often involve the stomach [5]. In his *Theory of the Spleen and Stomach*, Dongyuan Li also pointed out that “the internal injury to the spleen and stomach is caused by all diseases,” saying, “If the stomach qi is weak and the diet is doubled, then the qi of the spleen and stomach is injured, and the yuan qi cannot be filled, and all diseases are caused.” Traditional Chinese medicine (TCM) regards the spleen and stomach as the acquired foundation, the hub of yin and yang, and the enrichment of the spleen qi and stomach qi is the basis for the normal function of the viscera. All diseases are caused by the spleen and stomach. If the spleen and stomach are damaged, the yuan qi is damaged. If the yuan qi is lost, the yang qi is the first to be consumed, causing deficiency and cold inside [6]. Therefore, clinically, most spleen and stomach diseases are caused by deficiency cold syndrome. Spleen and stomach deficiency and cold stomach pain are often symptoms of overall cold and deficiency. The main manifestations are cold stomach and stomach pain aggravated by an empty stomach, relief of stomach pain after stomach massage and hot compresses, mental fatigue, weakness of the limbs, diarrhea, decreased taste in the mouth, lack of thirst, cold limbs, a layer of white material on the tongue surface and a fat tongue resulting in teeth marks on both sides of the tongue pressed inward, and slow and weak pulse. The treatment in principle should be based on warming the spleen, dispersing cold, and relieving pain [7].

At present, quadruple therapy of conventional Western medicine for treating spleen and stomach diseases includes inhibition of gastric acid secretion, protection of gastric mucosa, and two antibiotic treatments, which has become the first choice for the treatment of spleen and stomach diseases because of their fast onset and a short course of treatment. However, the fast onset of Western medicine not only causes side effects of the drugs but also gradually generates drug resistance in the human body [8], which increases the burden on the liver and kidneys. In addition, Western medicine only cures the exterior of the spleen and stomach deficiency cold without treating the root of the disease, which leads to recurrence. With the continuous improvement of quality of life, people are increasingly eager for alternative therapy with fewer toxic side effects and effective treatment for the pain caused by the spleen and stomach cold.

TCM is a treasure of world civilization with a long history of more than 5000 years. TCM methods, such as moxibustion, acupuncture, herbal acupuncture point application, and hot package therapy, are widely used in the treatment of various diseases. Moxibustion has a unique status in TCM treatment, and moxibustion therapy is widely used in the treatment of spleen and stomach deficiency cold-type stomach pain, which is popular among patients because of its unique advantages, including fast speed, stable long-term effects, multipath balanced regulation, and high total efficiency [9].

Moxibustion is a commonly used external treatment in Chinese medicine [10], in which mugwort or moxa leaves (Folium Artemislae Argyi) are made into strips and then ignited to put directly or indirectly on the relevant areas, and its warming stimulation and drug effects have unique therapeutic effects in the treatment and prevention of diseases and health care. The warming effect of moxibustion can increase the local blood speed, improving the local microcirculation and affecting the function of internal organs, especially those involved in gastrointestinal microcirculation [11]. One study showed that moxibustion can alleviate low-grade gastrointestinal inflammation in postinfectious/postinflammatory IBS (PI-IBS) and relieve visceral hypersensitivity by regulating intestinal microorganisms and controlling NLRP6 inflammatory small body signal conduction, thus controlling gastrointestinal inflammation and regulating gastrointestinal movement [12]. Therefore, moxibustion can fundamentally treat spleen and stomach deficiency and cold stomach pain.

Currently, moxibustion combined with Chinese medicine is widely used in the treatment of spleen and stomach deficiency cold-type stomach pain; however, its therapeutic effect is still controversial. In a study on gastrointestinal diseases, some scholars questioned the effectiveness of moxibustion in treating gastric diseases [13]. Therefore, the aim of this study was to pool all relevant randomized controlled trials and provide a clinical basis for the treatment of patients with spleen and stomach deficiency cold-type gastroparesis with moxibustion combined with TCM through systematic evaluation and meta-analysis.

## 2. Methods

### 2.1. Study Profile

We prospectively registered the protocol on the PROSPERO platform (PROSPERO (York.ac.uk) under the registration number CRD42022321692.

### 2.2. Search Strategy

The study was conducted according to the guidelines of the Preferred Reporting Item for Systematic Reviews and Meta-Analyses (PRISMA) statement [14]. A computer search was conducted by 2 researchers (search, selection, and analysis of the studies by the first and second authors independently), and 8 databases and two clinical trial centers were included in the search: Web of Science, PubMed, Embase, the Cochrane Library, China National Knowledge Infrastructure (CNKI), Wan-fang Data, China Science and Technology Journal Database (VIP), the Chinese Biomedical Literature Database (CBM), Clinical Trials.gov, and Chinese Clinical Trial Registry. All published documents, conference journals, and unpublished studies were searched. After pre-examination, we retrieved all publicly published literature thus far, and retrieved words in accordance with the Cochrane Handbook, adopted an advanced retrieval strategy combining subject words and free words, used the synonym extension function, adjusted the retrieval strategy in accordance with the corresponding database, and determined the final retrieval strategy. The search strategy contained the following 3 core components, which were linked using “AND”: (a) moxibustion (e.g., TCM moxibustion, moxibustion strip, TCM treatment with moxibustion); (b) spleen and stomach deficiency cold-type gastroparesis; and (c) randomized controlled trial (e.g., randomized, placebo). A comprehensive PubMed retrieval strategy can be obtained from the Supplementary materials “(available (here))” at Hindawi SSO.

### 2.3. Inclusion and Exclusion Criteria

#### 2.3.1. Inclusion Criteria were Developed According to the PICOS Framework [15]

*Participants*. Patients were diagnosed with spleen and stomach deficiency cold-type gastroparesis by diagnostic criteria; that is, a gastroscopy was diagnosed as stomach disease [16], in accordance with the diagnostic criteria of spleen and stomach deficiency cold-type stomach pain in the Consensus Opinions on Integrated Chinese and Western Medicine Treatment of Chronic Gastritis [17]. The main symptoms were (1) pain in the stomach and epigastric region and (2) relief of stomach pain after stomach massage and hot compresses. Secondary symptoms included (1) stomach fullness after eating; (2) decreased appetite; (3) diarrhea; (4) self-conscious fatigue; and (5) a layer of white material on the tongue surface and a fat tongue resulting in teeth marks on both sides of the tongue pressed inward. Gastroscopic images show (1) thin and abundant mucus; (2) reduced inflammation of gastric mucosa or pale, thinning mucosa; and (3) slow gastric peristalsis. The diagnosis was confirmed when a patient had 2 main symptoms plus 1 secondary symptom or 1 main symptom plus 2 secondary symptoms combined with gastroscopic signs.

*Interventions*. The experimental group received moxibustion therapy or moxibustion therapy supplemented with other Chinese medical methods (Chinese medicine includes acupuncture, tonics, Tuina, massage, acupuncture point compress, Chinese medicine heat package, and diet therapy.).

*Controls*. The control group was given regular Western medical treatment, including inhibition of gastric acid secretion, protection of gastric mucosa, and eradication of *Helicobacter pylori*.

*Outcomes*. The outcomes included an effective rate of evaluation of TCM outcome indicators or Western medicine outcome indicators.

1. TCM outcome indicators: referring to the consensus opinions of experts on the diagnosis and treatment of epigastric pain published in the Journal of Traditional Chinese Medicine in 2017 [18], we used the following indicators to judge the effectiveness of the treatment of spleen and stomach deficiency cold-type gastroparesis: (1) cured: symptoms and signs disappeared or basically disappeared, and the symptom score decreased by ≥ 95%; (2) good: symptoms and signs improved significantly, and symptom score decreased by ≥70%–95%; (3) effective: symptoms and signs improved, and the symptom score decreased by ≥ 30%–70%; and (4) ineffective: symptoms and signs did not improve significantly or even worsened, and the symptom score decreased by < 30% or increased. The following calculation formula (Nimodipine method) was used: symptom efficacy = (pretreatment points − posttreatment points)/pretreatment points × 100.00%.
2. The Western medicine outcome index [19] was as follows: (1) cured: gastroscopy showed normal results, the patient's clinical symptoms had disappeared, and the stomach was free of inflammatory reactions and gastric ulcers; (2) effective: gastroscopy showed improvement, the patient's clinical symptoms were significantly improved, the inflammatory reaction in the stomach was reduced, and the extent of gastric ulcer was reduced; and (3) invalid: gastroscopy showed invalid results, the patient's inflammatory reaction and ulcer scope did not improve or became visibly aggravated, and clinical symptoms did not improve significantly. The total effective rate was calculated as = (number of cured cases + number of effective cases)/total number of cases × 100%.

*Studies.* All randomized controlled studies published nationally and internationally were included.

The exclusion criteria were as follows: (a) duplicate published studies; (b) studies for which the full text was not available; (c) studies for which valid data could not be obtained; (d) studies involving animal tests, systematic evaluations, and reviews; and (e) professional aspects: (1) patients taking other therapeutic gastric drugs in the last two weeks; (2) patients with severe combined gastrointestinal diseases, such as combined hypertension and hyperthermia; (3) pregnant women or those with mental abnormalities; (4) patients with broken skin at the application site or those with contraindications; and (5) patients with serious gastric diseases such as gastric tumors.

### 2.4. Screening and Data Extraction

The retrieved studies were imported into EndNote file management software, and two researchers (the first reviewer and second reviewer) independently screened the studies according to the identified inclusion criteria and extracted the literature information. They cross-checked the results from each step, and any inconsistencies were settled through discussion or by input from the third reviewer. Two authors read the titles and abstracts for initial screening after eliminating duplicates and browsed the full text of the remaining studies to determine the final included studies based on the determined standards. Extracted information included authors, year of publication, sample size, gender, age, interventions, efficiency, and outcome indicators. Excel software was used to extract and record the literature information. If any data were incomplete or missing, we contacted the authors by phone or e-mail to obtain more comprehensive data information, and if comprehensive information could not be obtained by the above means, the study was excluded.

### 2.5. Quality Evaluation

The methodological quality of the RCTs was assessed and cross-checked by two systematically trained postgraduates (Qingwen Gan and Zerong Lian) and, in cases of disagreement, adjudicated in consultation with a third party (Lilan Zheng). We evaluated the risk of bias associated with each study using the revised Cochrane risk-of-bias tool for randomized trials (RoB 2) [20]. The RoB 2 evaluation tool contains the following 5 evaluation domains: (1) randomization process; (2) deviations from intended interventions; (3) missing outcome data; (4) measurement of the outcome; and (5) selection of the reported result. There are multiple different signal questions under each domain, and the signal questions have five choices for answers “Y (Yes),” “PY (Probably Yes),” “PN (Probably No),” “N (No),” and “NI (No Information).” The domain of bias by deviation from the established intervention was divided into two options according to different research purposes: one was to study the effect of the intervention assignment, and the other was the effect of intervention adherence.

### 2.6. Statistical Analysis

Excel software was used to extract and record study information, and the statistical software used was Review Manager 5.4 and Stata 17.0 for meta-analysis. The heterogeneity of the included studies was analyzed by the *Q*-test (test level *α* = 0.05), and the size of heterogeneity was quantified by combining *I*^2^: If *P* > 0.01 and *I*^2^ < 50%, the data were combined using a fixed effects model (FEM); otherwise, a random effects model (REM) was applied. When *I*^2^>50% indicated significant heterogeneity, studies were subjected to subgroup analysis or sensitivity analysis. Sensitivity analysis is a method to evaluate the stability of meta-analysis results by excluding the included studies one by one; subgroup analysis divides the study subjects into different subgroups according to certain characteristics (gender, disease severity, etc.) to reduce heterogeneity between groups. The test level of the meta-analysis was *α* = 0.05, the odds ratio (OR) or RR was chosen as the combined effect size for dichotomous data, and each effect size was expressed as the 95% confidence interval (CI). Publication bias was tested by funnel plot; better symmetry of the funnel plot suggested less publication bias, and the symmetry of the funnel plot was tested by the Egger test.

## 3. Results

### 3.1. Search Results

**Figure 1:**
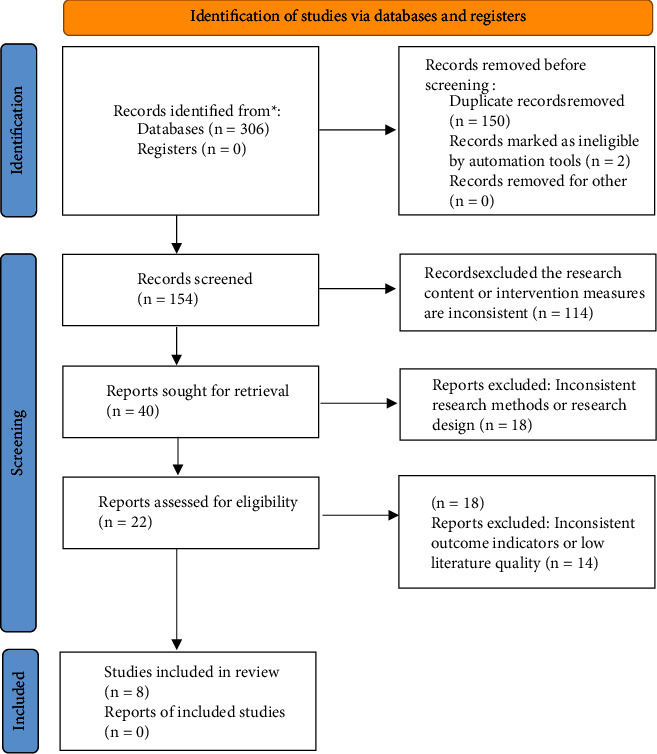
Diagram of the study selection process.

A total of 306 papers were retrieved, 296 in Chinese and 10 in English; 150 duplicates were excluded by using Endnote literature management software, and 2 papers were excluded by an online search for animal testing, systematic evaluation, and review papers. After reading the titles and abstracts of the papers, 114 papers that did not match the research content and intervention measures were excluded. The remaining 40 papers were read in full and rescreened to further exclude 32 papers, and 8 papers were finally included [21–28]. The literature screening process is shown in Figure 1.

### 3.2. Characteristics of Included Studies

Eight RCT studies [21–28] published between 2012 and 2021 were included, with a total of 729 patients with spleen and stomach deficiency cold-type gastroparesis, of whom the youngest was approximately 24 years old, the oldest was approximately 71 years old, the mean age was approximately 48 years, the longest duration of disease was 8 years and the shortest was 17 months, and there was no significant difference in the proportion of men and women in the study population. Interventions included moxibustion therapy, Sanhe decoction, acupoint application, *Astragalus* Jianzhong soup, TCM package, and discriminative food therapy, and the measured outcome indicators included TCM evidence score, gastroscopy, and VAS. The basic characteristics of the included studies are shown in Table 1.

### 3.3. Methodological Evaluation of the Included Studies

**Figure 2:**
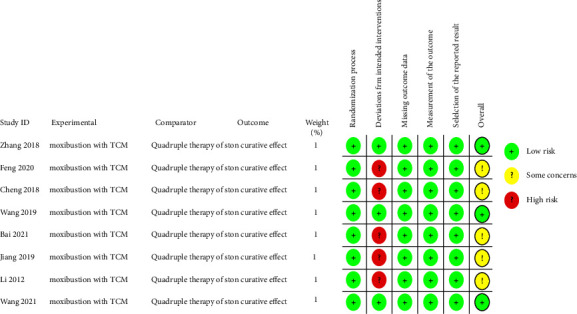
Risk-of-bias graph.

**Figure 3:**
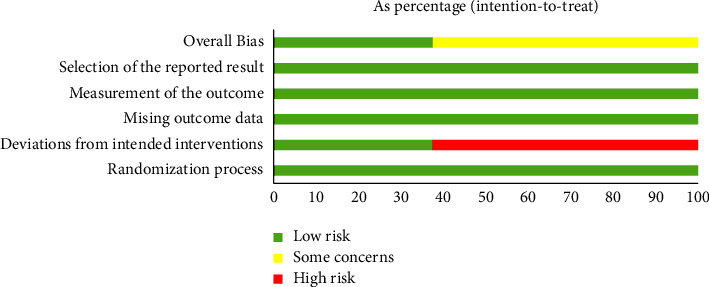
Risk-of-bias summary.

The eight included studies analyzed the comparability of the patient's age, sex, course of the disease, and other baseline data, and the difference was not statistically significant (*P* > 0.05). Three of the included studies [21, 24, 25] mentioned signing informed consent with patients, and five studies [22, 23, 26–28] did not report whether participants were blinded. Regarding the generation of random sequences, three studies [24, 25, 27] are mentioned using a random number table method, and five studies [21–23, 26, 28] did not report the generation of random sequences by any specific method. Using the RoB 2 assessment tool to assess the risk of bias in the included studies, all eight papers [21–28] had a low risk of bias for the “randomization process,” “missing outcome data,” “measurement of the outcome,” and “selection of the reported result”; 5 papers [21–23, 26, 28] were at high risk of bias in “deviations from intended interventions,” and the risk of bias was low in three papers [24, 25, 27]. The overall risk of the included studies generated some concerns because of the specificity of randomized controlled trial interventions, which are difficult to blind during implementation and therefore cannot avoid the risk of deviating from the established interventions. The methodological evaluation of the studies is shown in Figures 2 and 3.

### 3.4. Meta-Analysis Results of Included Studies

#### 3.4.1. Efficacy Index

According to clinical practice, the categories of cured, good, and effective were combined into an effective category and the rest were combined into an ineffective category. The effective rate between the research groups was compared and merged, and the efficacy of each study is shown in Table 2.

#### 3.4.2. Effective Rate of Moxibustion with TCM

**Figure 4:**
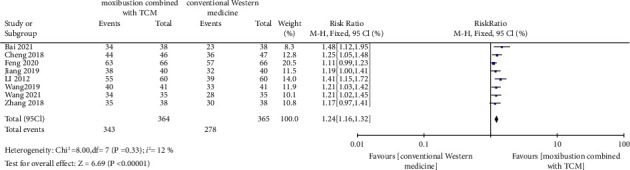
Forest plot.

The meta-analysis heterogeneity test results of *I*^2^ = 12% and *P*=0.33 indicated that there was no significant heterogeneity in this study, and a fixed effects model was selected for statistical analysis. The RR value of the combined pooled effect size of the eight studies was 1.24 (95% CI 1.16–1.32), and statistical significance test results of *Z* = 6.69 and *P* < 0.05 suggested that moxibustion combined with TCM was more effective in treating spleen and stomach deficiency and cold stomach pain than conventional Western medicine. The details are shown in the forest plot in Figure 4.

#### 3.4.3. Sensitivity Analysis

**Figure 5:**
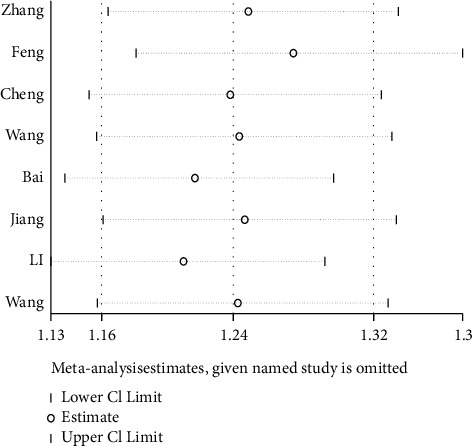
Sensitivity analysis.

To ensure the stability of the study results sensitivity analysis was performed on the eight included studies using the exclusion-by-exclusion method, and the results of the sensitivity analysis showed that the combined RR value after excluding any one study was 1.24 with a 95% CI (1.16–1.32) similar to the total combined effect value, suggesting that the results of this study are stable and credible. The results are shown in Figure 5.

#### 3.4.4. Publication Bias

**Figure 6:**
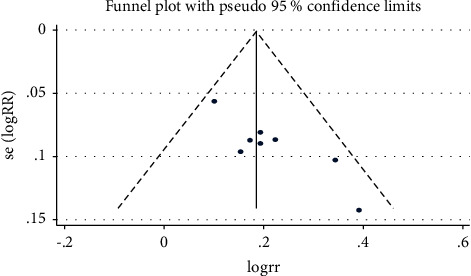
Funnel plot.

A funnel plot was drawn to determine whether this study had publication bias, and the symmetry of the funnel plot, as tested for publication bias by Egger's test (*P* > 0.05), suggested that this study did not have publication bias.  Figure 6 shows the funnel plot.

### 3.5. Safety

Only four RCTs had information on moxibustion with TCM-related adverse events [21, 22, 26, 28], of which two mentioned that no adverse events occurred [21, 28]. In the other two randomized controlled trials, one study [22] reported mild discomfort in one patient after moxibustion with TCM, and the other study [26] reported that there were few adverse reactions after moxibustion with TCM treatment.

## 4. Discussion

### 4.1. Strengths and Limitations

The evidence pooled in this study demonstrates that moxibustion combined with Chinese medicine is more effective than conventional Western medicine in treating spleen and stomach deficiency cold-type gastroparesis, but this study still has limitations. The methodological quality of the included articles was not high, and there was a risk of bias, which lowered the level of evidence. Future studies need to adopt a more rigorous design to ensure reasonable random sequence generation, allocation concealment, and blinding. Adverse events such as burns and follow-up of moxibustion treatment were not described in the included studies; it is recommended that moxibustion treatment be performed by professional acupuncturists in strict accordance with the criteria and that a moxibustion apparatus be used during the operation to reduce the occurrence of adverse events such as burns. Follow-up of patients treated with moxibustion combined with Chinese medicine can be increased in future studies to provide a clearer understanding of the effect of treatment.

This systematic evaluation also has many strengths. First, after a computer search, it was found that there was no meta-analysis related to moxibustion combined with TCM to treat spleen and stomach deficiency and cold stomach pain. This study is the first of its kind and summarizes all existing published and unpublished relevant studies based on clinical practice and patient needs. The literature provides systematic clinical evidence that moxibustion combined with TCM is a potential treatment for patients with spleen and stomach deficiency and cold stomachache, and it improves the quality of life of patients. Second, this study conducted a prior search, analysis, primary screening, and literature quality evaluation of relevant studies on moxibustion combined with Chinese medicine for the treatment of spleen and stomach deficiency cold-type gastroparesis using existing databases and clinical trial registries to make the study more rigorous. Furthermore, we had two investigators conduct the analysis independently to improve the rigor of the study, and in the process of including studies, we strictly followed the nadir criteria for screening. Studies with incomplete information were contacting the authors who did not obtain more information, duplicate publications, studies with ambiguous literature information, and studies with inconsistent outcome indicators were excluded. The whole process of the meta-analysis was open, transparent, and repeatable, which improved the transparency of the study.

### 4.2. Summary of Evidence

This study includes eight RCT studies that conform to our standards, and the efficacy of moxibustion combined with Chinese medicine for the treatment of spleen and stomach deficiency cold-type gastroparesis was finally concluded to be superior to conventional Western medicine by pooling the effect size RR and calculating the 95% CI and tested for heterogeneity (*I*^2^ = 12% <50%), sensitivity analysis (using the reject-by-article method), and publication bias (funnel plot and Egger's test method). The difference was statistically significant (*P* < 0.05). This study is consistent with the findings of Han et al. [29] *in Chinese Medicine Modern Distance Education of China*, who mentioned the clinical observation of moxibustion combined with a Chinese medicine acupoint patch in the treatment of patients with spleen and stomach deficiency cold-type stomach pain, Jiang Lei et al. [30] in the clinical observation of acupuncture combined with moxibustion in the treatment of spleen and stomach deficiency cold-type stomach pain, and Ru and Sun et al. [31] in the clinical observation of moxibustion in the treatment of spleen and stomach deficiency cold-type stomach pain, which provides a clinical basis for the future treatment of patients with spleen and stomach deficiency cold-type stomach pain.

### 4.3. Mechanism of Moxibustion Treatment

Modern research has confirmed that moxibustion creates far and near-infrared radiation and heat and light radiation. Far infrared radiation penetrates the internal organs and meridians and acts directly on the focal area, which can penetrate into the circulation and nervous system and be absorbed by living tissues to achieve therapeutic effects [32], so moxibustion is widely used in the treatment of various diseases [33–35]. According to the theory of Chinese medicine, moxibustion therapy is applied to certain acupuncture points of the human body through moxa wool or other ignited drugs so that its heat and medicinal effects can penetrate the skin to warm the meridians, dispel cold and dampness, and unblock the meridians to regulate the body's qi and blood [36]. The body's qi and blood transport the nutrients in food, moisten the internal organs and harmonize the spleen and stomach when they run smoothly. The Yin and Yang balance [37] is supplemented with other Chinese medicine methods to effectively treat spleen and stomach deficiency cold-type of stomach pain.

Similar to the Western medicine mechanism, moxibustion can stimulate the receptors and afferent nerves on the meridians connected to acupuncture points to produce nerve impulses; then, through the reflexes and conduction of the nervous system, it can regulate the physiological activities and pathological changes of internal organs and tissues, and enhance immunity and resistance to disease to achieve disease prevention and treatment. Some scholars [38, 39] pointed out that moxibustion can play a role in the repair of acute gastric mucosal injury by affecting the vagus nerve. Zusanli (ST36) can initiate the endogenous protective information transmission process through the common peroneal nerve pathway to achieve a protective effect on the gastric mucosa. In animal experiments, some researchers [40, 41] found that moxibustion at the Zusanli (ST36) and Zhongwan (CV12) acupoints could increase the expression of EGF and transforming growth factor alpha (TGF-*α*) in the gastric tissues of rats with acute gastric mucosal injury while decreasing the content of triglyceride family 3 (TFF3) in the gastric mucosa to protect the gastric mucosa. Moxibustion at the corresponding acupoints could participate in neuroendocrine regulation, thus inhibiting the hyperactive hypothalamic-pituitary-adrenal (HPA) axis of rats in the restraint-submersion model, which can fundamentally control the extent of gastric mucosal damage by improving the stress state. In addition, it has also been found [42, 43] that moxibustion has a modulating effect on metabolites such as glutathione, N-acetylnucleotide, phosphorylcholine, and uracil in CAG rats, suggesting a specific relationship between the gastric meridian and the stomach. Moxibustion treatment regulated the expression of EGF and EGFR, and the metabolites (alanine, nicotinamide adenine dinucleotide phosphate, uracil DNA glycosylase, lactate, glycerol, and adenosine) induced by CAG in gastric tissues were restored to normal levels after moxibustion treatment, thus reducing the apoptosis of gastric mucosal cells and controlling acute and chronic gastric mucosal damage to treat spleen and stomach diseases.

### 4.4. Clinical Significance for Future Studies

As a noninvasive treatment, moxibustion adjusts the physiological functions of the body through meridians, promotes metabolism, enhances blood circulation, improves gastrointestinal function, and increases the immunity and disease prevention ability of the body. At the same time, it is a simple and easy low-risk economic activity without toxic side effects; therefore, we recommend promoting the use of moxibustion therapy in the public health system, and patients and their families can perform it at home at any time after guidance and practice with professional acupuncturists to achieve the effect of curing and preventing diseases. In addition, we suggest that future criteria for recruiting participants should be more specific and systematic to improve representation and avoid bias and that training programs for moxibustion therapy should be standardized to better explore the therapeutic effects of moxibustion in combination with TCM for patients with spleen and stomach deficiency cold-type gastroparesis.

## 5. Conclusion

According to the results of systematic evaluation, the efficacy of moxibustion combined with TCM in the treatment of spleen and stomach cold stomach pain is better than that of conventional Western medicine. In future treatments of patients with spleen and stomach cold stomach pain, moxibustion can be given to patients according to their clinical indications and supplemented with other TCM therapy to reduce the stimulation of gastric mucosa by internal medicine, alleviate the pain and improve the quality of life of patients. However, the quality level of the studies included in this paper is not high, and there is a potential risk of bias, so more high-quality, large-sample, multicenter randomized controlled studies on moxibustion with TCM for the treatment of spleen and stomach deficiency cold-type gastroparesis at home and abroad are needed in the future to improve the reliability of the study results.

## Figures and Tables

**Table 1:** Characteristics of the included studies.

Study (author/year)	Sample size	Gender (male/female)	Age (*X* ± *S*) (*E*/*C*)	Intervention measures	Outcome indicators	Efficient (%) (*E*/*C*)
Zhang 2018	76 (*E* = 38/*C* = 38)	*E* (16/22) *C* (17/21)	*E* (42.8 ± 11.2) *C* (42.5 ± 10.3)	*M* + SHD	TCMSS	92.1/78.9
Feng 2020	132 (*E* = 66/*C* = 66)	*E* (30/36) *C* (31/35)	*E* (45.34 ± 6.25) *C* (43.17 ± 6.73)	*M* + AA	VAS + *G*	95.5/86.4
Chen 2018	93 (*E* = 46/*C* = 47)	*E* (16/30) *C* (18/29)	*E* (41.03 ± 17.48) *C* (40.32 ± 18.56)	*M* + AJZS	*G*	95.7/76.6
Wang 2019	82 (*E* = 41/*C* = 41)	*E* (21/20) *C* (19/22)	*E* (45.2 ± 11.8) *C* (47.1 ± 12.3)	*M* + AA	TCMSS + *G* + VAS	97.56/80.49
Bai 2021	76 (*E* = 38/*C* = 38)	*E* (17/21) *C* (20/18)	*E* (43.45 ± 8.2) *C* (43.36 ± 8.70)	*M* + AJZS	TCMSS + *G*	89.47/60.53
Jiang 2019	80 (*E* = 40/*C* = 40)	*E* (29/11) *C* (27/13)	*E* (35.36) *C* (34.36)	*M* + TCP	TCMSS	95.0/80.0
Li 2012	120 (*E* = 60/*C* = 60)	*E* (28/32) *C* (29/31)	*E* (50.5) *C* (49.0)	*M*	TCMSS	91.67/65.0
Wang 2021	70 (*E* = 35/*C* = 35)	*E* (18/17) *C* (20/15)	*E* (68.1 ± 2.4) *C* (54.8 ± 2.1)	*M* + DT	TCMSS + VAS	97.14/80.00

*E* = experience group; *C* = control group; *M* = moxibustion; SHD = Sanhe decoction; AA = acupoint application; AJZS = *Astragalus* Jianzhong soup; DT = diet therapy; TCP = TCM package; *G* = gastroscope; VAS = visual analog scale; TCMSS = TCM syndrome score.

**Table 2:** Efficacy of clinical studies.

Author	Year	Sample size (experience group)		Sample size (control group)		Efficient (%) (experiment/control)
		Valid	Total	Valid	Total	
Zhang	2018	35	38	30	38	92.1/78.9
Feng	2020	63	66	57	66	95.5/86.4
Chen	2018	44	46	36	47	95.7/76.6
Wang	2019	40	41	33	41	97.56/80.49
Bai	2021	34	38	23	38	89.47/60.53
Jiang	2019	38	40	32	40	95.0/80.0
Li	2012	55	60	39	60	91.67/65.0
Wang	2021	34	35	28	35	97.14/80.00

## Data Availability

The data supporting this meta-analysis are from previously reported studies and datasets, which have been cited. The processed data are available from the corresponding author upon request.

## Supplementary Materials

Supplementary Materials The complete search expression can be obtained in the submission system as supplementary materials.()
