# Supplementary material for: Effectiveness of Moxibustion Combined with Chinese Medicine in the Treatment of Spleen and Stomach Deficiency Cold-Type Gastroparesis: A Meta-Analysis of Randomized Controlled Trials
Source: Evid Based Complement Alternat Med. 2022 Nov 10;2022:6552819. doi: 10.1155/2022/6552819 (PMC11401712; doi:10.1155/2022/6552819)
Supplement: Supplementary Materials — The complete search expression can be obtained in the submission system as supplementary materials.() [file 6552819.f1.docx]

1. **PubMed Search (0)**

#1 "Moxibustion"[Mesh]

#2 (((moxibustion[Title/Abstract]) OR (Traditional Chinese medicine moxibustion[Title/Abstract])) OR (Moxibustion strip[Title/Abstract])) OR (Traditional Chinese medicine treatment with Moxibustion[Title/Abstract])

#3 ("Moxibustion"[Mesh]) OR ((((moxibustion[Title/Abstract]) OR (Traditional Chinese medicine moxibustion[Title/Abstract])) OR (Moxibustion strip[Title/Abstract])) OR (Traditional Chinese medicine treatment with Moxibustion[Title/Abstract]))

#4 “Spleen and Stomach Deficiency Cold-Type Gastroparesis” [Title/Abstract]

#5 randomized controlled trial[Publication Type] OR randomized[Title/Abstract] OR placebo[Title/Abstract]

#6 **#3 AND #4 AND** #5


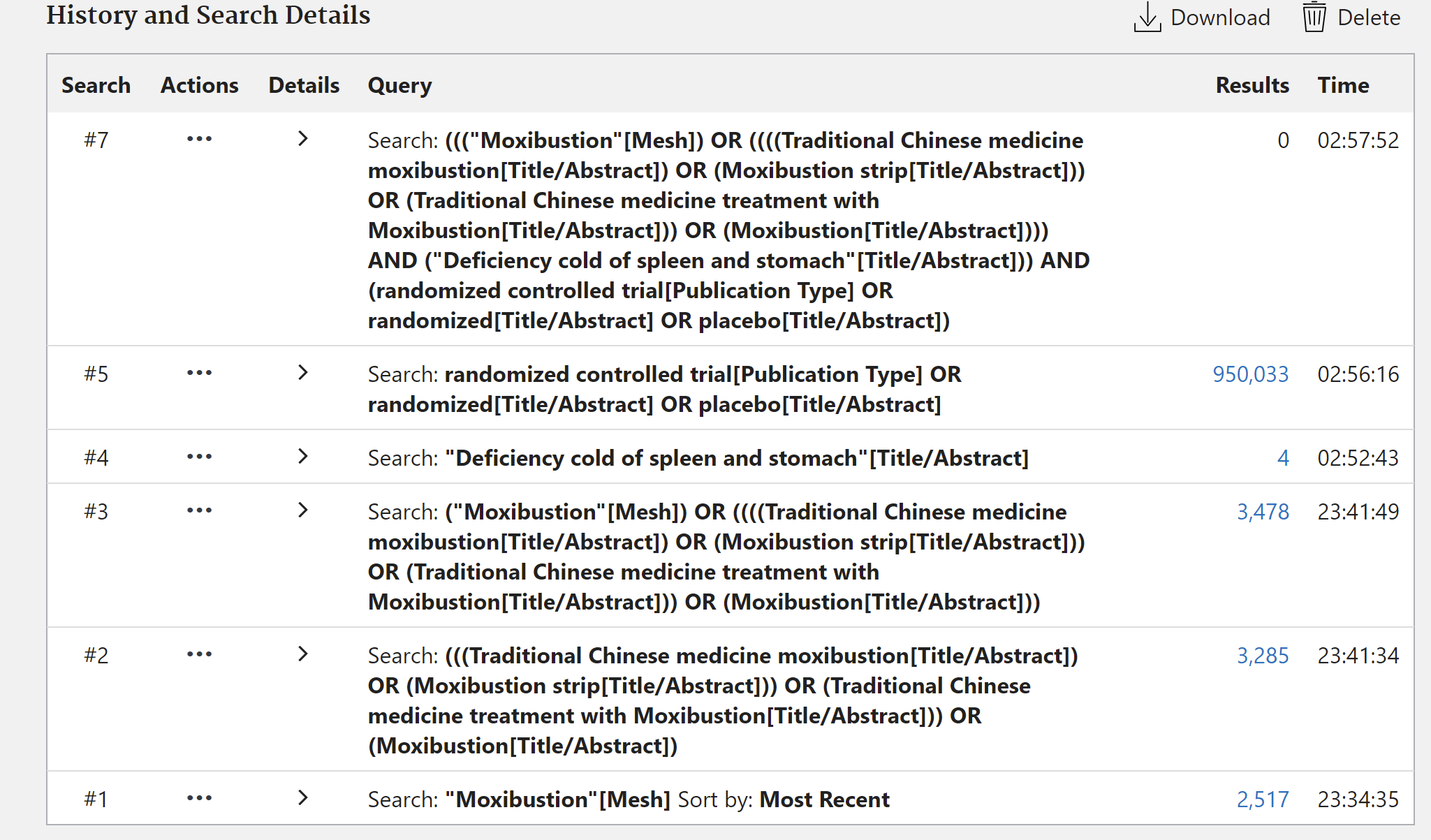


**2．Web of science(2)**

#1 **(((TS=(Moxibustion strip)) OR TS=(Moxibustion )) OR TS=(Traditional Chinese medicine treatment with Moxibustion)) OR TS=(Traditional Chinese medicine moxibustion)**

**#2 (((((TS=(Epigastric disease of spleen stomach deficiency cold type)) OR TS=(Deficiency cold of spleen and stomach)) OR TS=(Spleen stomach deficiency cold type)) OR TS=(Spleen stomach deficiency cold gastritis)) OR TS=(Spleen stomach deficiency cold stomach disease)) OR TS=(Spleen stomach deficiency cold stomach pain)**

#3 **(((TS=(randomized controlled)) OR TS=(randomized controlled trial)) OR TS=(random)) OR TS=(RCT)**

#4 #1 AND #2 AND #3

**3、Embase(0)**


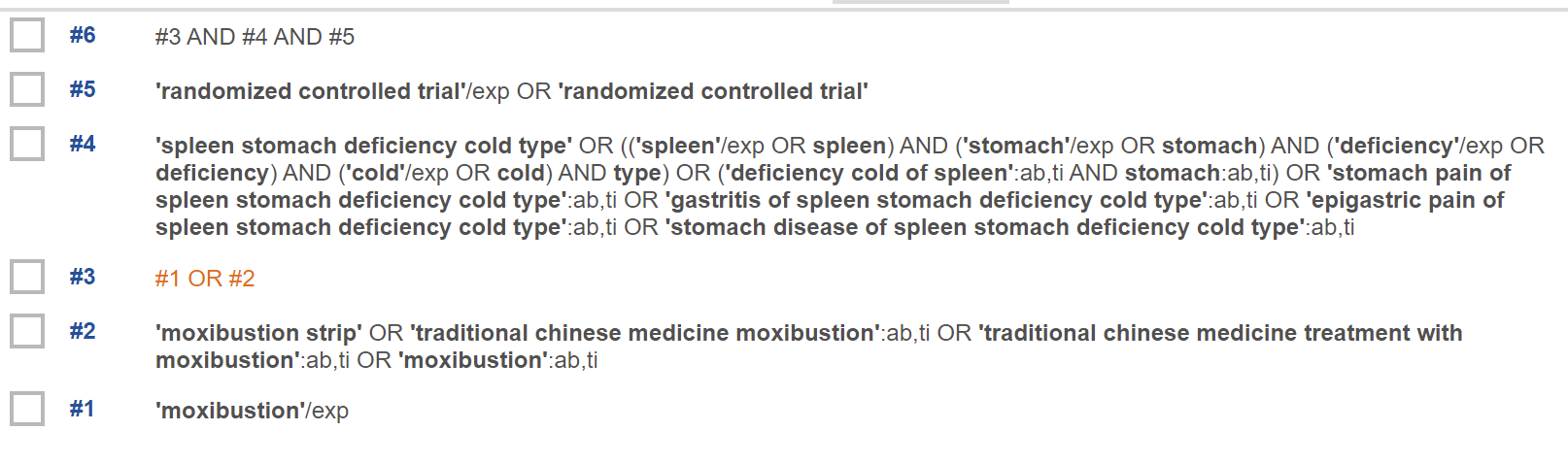


**4. Ccochrane（8）**

#1 MeSH descriptor: [Moxibustion] explode all trees

#2 (Moxibustion strip):ti,ab,kw OR (Traditional Chinese medicine treatment with Moxibustion):ti,ab,kw OR (Traditional Chinese medicine moxibustion):ti,ab,kw OR (Moxibustion):ti,ab,kw

#3 #1 or #2

#4 (Spleen stomach deficiency and cold stomach):ti,ab,kw OR (Spleen stomach deficiency cold type):ti,ab,kw OR (Gastritis of spleen stomach deficiency cold type):ti,ab,kw OR (Epigastric pain of spleen stomach deficiency cold type):ti,ab,kw OR (Stomach pain of spleen stomach deficiency cold type):ti,ab,kw

#5 #3 and #4
